# Exploring women’s interpretations of survey questions on pregnancy and pregnancy outcomes: cognitive interviews in Iganga Mayuge, Uganda

**DOI:** 10.1186/s12978-024-01745-w

**Published:** 2024-01-29

**Authors:** Doris Kwesiga, Mats Malqvist, Christopher Garimoi Orach, Leif Eriksson, Hannah Blencowe, Peter Waiswa

**Affiliations:** 1 Department of Women’s and Children’s Health, Uppsala University, Uppsala, Sweden; 2 Department of Health Policy, Planning and Management, School of Public Health, Makerere University, Kampala, Uganda; 3 Department of Community Health and Behavioral Sciences, School of Public Health, Makerere University, Kampala, Uganda; 4 Maternal, Adolescent, Reproductive and Child Health Centre (MARCH), London School of Hygiene and Tropical Medicine, London, UK; 5 Department of Global Public Health, Karolinska Institutet, Stockholm, Sweden; 6 Busoga Health Forum, Jinja, Uganda

**Keywords:** Cognitive interviews, Surveys, Demographic and Health Survey

## Abstract

**Background:**

In 2021, Uganda’s neonatal mortality rate was approximately 19 deaths per 1000 live births, with an estimated stillbirth rate of 15.1 per 1000 total births. Data are critical for indicating areas where deaths occur and why, hence driving improvements. Many countries rely on surveys like Demographic and Health Surveys (DHS), which face challenges with respondents’ misinterpretation of questions. However, little is documented about this in Uganda. Cognitive interviews aim to improve questionnaires and assess participants’ comprehension of items. Through cognitive interviews we explored women’s interpretations of questions on pregnancy and pregnancy outcomes.

**Methods:**

In November 2021, we conducted cognitive interviews with 20 women in Iganga Mayuge health and demographic surveillance system site in eastern Uganda. We adapted the reproductive section of the DHS VIII women’s questionnaire, purposively selected questions and used concurrent verbal probing. Participants had secondary school education and were English speaking. Cognition was measured through comparing instructions in the DHS interviewers’ manual to participants’ responses and researcher’s knowledge. A qualitative descriptive approach to analysis was undertaken.

**Results:**

We report findings under the cognitive aspect of comprehension. Some questions were correctly understood, especially those with less technical terms or without multiple sections. Most participants struggled with questions asking whether the woman has her living biological children residing with her or not. Indeed, some thought it referred to how many living children they had. There were comprehension difficulties with long questions like 210 that asks about miscarriages, newborn deaths, and stillbirths together. Participants had varying meanings for miscarriages, while many misinterpreted stillbirth, not linking it to gestational age. Furthermore, even amongst educated women some survey questions were misunderstood.

**Conclusions:**

Population surveys may misclassify, over or under report events around pregnancy and pregnancy outcomes. Interviewers should begin with a standard definition of key terms and ensure respondents understand these. Questions can be simplified through breaking up long sentences, while interviewer training should be modified to ensure they thoroughly understand key terms. We recommend cognitive interviews while developing survey tools, beyond basic pre-testing. Improving respondents’ comprehension and thus response accuracy will increase reporting and data quality.

**Supplementary Information:**

The online version contains supplementary material available at 10.1186/s12978-024-01745-w.

## Background

Annually, a large number of newborn deaths and stillbirths are reported worldwide. In 2021, a report estimated global newborn deaths at 2.3 million per year, with a Neonatal Mortality Rate (NMR) of 18 deaths per 1000 live births [1]. Sub-Saharan Africa had the highest regional NMR at 27 deaths per 1000 live births while Uganda’s NMR was approximately 19 deaths per 1000 live births [1]. Key findings of the 2022 Uganda Demographic and Health Survey reported the country’s NMR at 22 deaths per 1000 live births [2]. In 2021, the global stillbirth rate was about 13.9 per 1000 total births, with the highest rate in sub-Saharan Africa [3]. The same report estimated Uganda’s stillbirth rate at 15.1 per 1000 total births in 2021 (based on 25,855 stillbirths per year).

Data are critical because they indicate areas where deaths are occurring, why, and associated challenges. Indeed, data are important to drive improvements in health and are a prerequisite for planning and for interventions at government and local government levels, in addition to their use by researchers, projects, civil society, international mortality estimates and more. However, countries with high numbers of Adverse Pregnancy Outcomes (APOs) frequently also have sub-optimal functioning of civil registration and vital statistics systems [4].

As a result, many countries, especially Low and Middle Income Countries (LMIC) rely on surveys like the Demographic and Health Surveys (DHS) for national data on pregnancy and APOs [5]. The DHS studies usually conducted every five years, are national and focused on key health, population and nutrition indicators. The standard DHS has four tools, namely; woman, man, household and biomarker questionnaires, in addition to collection of geographic information and standardized topics of interest for certain countries.

For data to be of good quality and beneficial though, respondents must clearly understand the questions asked, so they can give accurate answers. However, challenges have been identified with DHS data, for instance language of the tool, interviewer or respondent and whether a translator was involved [6]. Additionally, DHS is retrospective, asking about past events, hence prone to recall bias. Misclassification and omission of perinatal data are also reported challenges identified in these surveys [7] and response accuracy can be influenced by how the question is asked [4]. All these can impact the accuracy of data.

Among the approaches suggested to improve survey research is the use of cognitive interviewing, which has its roots in the cognitive sciences [8]. Various definitions of cognitive interviews exist, with a lack of consensus or standardization [9, 10]. One clear definition from Beatty and Willis (page 2) is that *cognitive interviewing entails administering draft survey questions while collecting additional verbal information about the survey responses, which is used to evaluate the quality of the response or to help determine whether the question is generating the information that its author intends* [9]. The focus of cognitive interviews was described by Willis and others as being on the survey questions and understanding the hidden and visible cognitive processes subjects, chosen according to specific traits of interest, undergo as they answer survey questions [11].

This process is founded on cognitive theory which has been represented by various frameworks, with the commonly used one developed by Tourangeau in 1984 [11]. Tourangeau’s framework broadly points out four key areas for the cognitive interview process: Comprehension, Retrieval, Decision and Response processes. Table 1 presents a summary of the four areas.

**Table 1:** Cognitive interview processes developed by Tourangeau

Focus area	Sub-components
Comprehension of questions	a) Question intent: What the respondent believes the question is asking b) Meaning of terms: What particular words mean to the respondent
Retrieval of information from memory	a) Recallability of information: The type of information the respondent needs to remember so as to answer the question b) Recall strategy: What strategies does the respondent use to retrieve information?
Decision processes	a) Motivation: Whether the respondent is applying adequate mental effort to provide thoughtful and accurate responses b) Sensitivity / social desirability: Whether the respondent is actually being truthful or simply wants to give answers that make him/her appear better
Response processes	Matching the response: Can respondent match his or her answer to the available response categories provided for the survey question?

Adapted from Willis GB. Cognitive Interviewing. A “How To” Guide. 1999 [11]

A range of approaches to cognitive interviewing have been highlighted in the literature but two broad ones are “think-aloud” interviewing and “verbal probing” techniques. In the think-aloud approach, the interview subject is requested to verbally express their thoughts as they respond to the survey questions, which expression can be concurrent or retrospective [12]. The verbal probing, on the other hand, involves the subject answering the question asked (without explaining their thought process aloud), after which the interviewer follows up with probes specific to the question or related to the answer given [11].

Cognitive interviews have been used in various ways to improve survey questions, communication and posters in different study areas and fields as diverse as health, nutrition and agriculture [13–17]. However, there was little published about women’s comprehension of DHS reproduction related questions in Uganda, more so from the DHS VIII version at the time this study was designed. It is unclear the extent to which questions are well understood and the subsequent potential effects on data quality. This study therefore explored women’s interpretations of selected DHS VIII questions in Iganga Mayuge Health and Demographic Surveillance System (HDSS) site, Uganda, through the use of cognitive interviews.

## Methods

### Study design

In November 2021, cognitive interviews were conducted with women in Iganga Mayuge HDSS (IMHDSS) run by Makerere University Center for Health and Population Research. This was a cross sectional study.

The current paper is part of a wider study nested within the EN-INDEPTH survey, which was a cross-sectional population based survey of 69,176 women of reproductive age, conducted between July 2017 and August 2018 in five HDSS sites (Bandim in Guinea Bissau, Dabat in Ethiopia, Iganga-Mayuge in Uganda, Matlab in Bangladesh and Kintampo in Ghana). The main EN-INDEPTH survey undertook a randomised comparison of the reproductive module used in the latest version of Full Birth History plus (FBH +) versus a Full Pregnancy History (FPH) module to examine the variation in capture of stillbirths and neonatal deaths. During the survey, the FPH approach identified more stillbirths in the community than the FBH + did [7]. Another study within EN-INDEPTH reported misclassification of stillbirths, with about a quarter found as born alive on further inquiry [18].

Knowledge learned through these and other previous EN-INDEPTH studies [19], for instance about questions that were problematic to women and which potentially influenced reporting of pregnancy and APOs, was used to further refine the current cognitive interviews. This study was conducted in IMHDSS due to physical accessibility for the researchers to do further in-depth work.

### Study setting

Set up in 2004, IMHDSS is found in the two districts of Iganga and Mayuge, in east central Uganda, covering 65 villages within 7 sub-counties and 155 kms squared. Two thirds of the HDSS is rural (with 51% engaged in agriculture), and the rest peri-urban or urban. In 2017, IMHDSS had 94,568 people in 18,634 households, with 48% of the members below 15 years old. As an open cohort, IMHDSS collects data bi-annually on basic demographics including births and deaths, in addition to doing verbal and social autopsies [20].

### Participant selection

Women whose education level was secondary school and above, within the age group of 15–49 years and living within the HDSS were purposively selected. This education level was specified because the interviews were to be conducted in English and by secondary school level in Uganda, students are able to speak and read English. Using both a purposive and convenience sampling approach, we worked with HDSS staff who went through their database and selected 20 women who matched our inclusion criteria, were living within proximity of the interview location in Iganga town where the interviews would be held and could participate in English interviews. One of the VHTs then assisted with mobilization of the selected respondents within the communities.

### Study tool

The study tool included an adaptation of the reproduction section of the women’s questionnaire in the DHS VIII [21]. Some questions on pregnancy and pregnancy outcomes were purposively selected from that questionnaire and used, without altering any of the wording. In this case, we were specifically exploring women’s comprehension of the questions and meaning of key terms in the questionnaire.

**Fig. 1:**
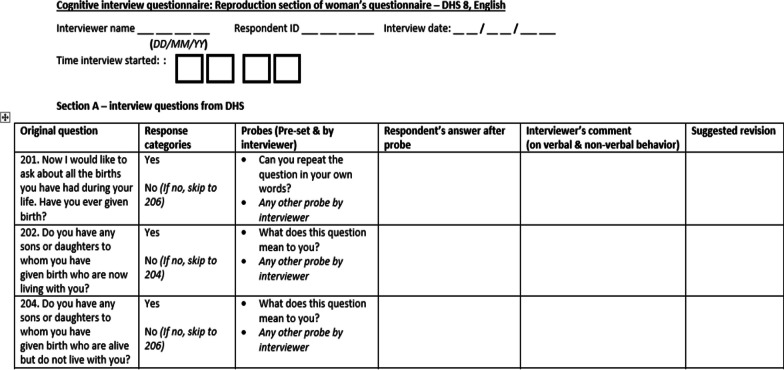
Excerpt of data collection tool

Additional sections of the tool included probes, respondents’ answers after probes, interviewer’s comments (on verbal and non-verbal behavior) and suggested revisions. An excerpt of the tool is shown in Fig. 1. Four research assistants received prior training on the tool and on conducting cognitive interviews. The complete tool is available in Additional file 1.

### Data collection

The verbal probing approach was used during this study because of familiarity with the method and we also assumed that women would be more comfortable with this approach than being asked to verbalize their thoughts for each response they had to make. More so, the study’s main focus was on comprehension of the questions and key terms, not the recall and decision aspects of cognition. We tried to avoid bias by ensuring our probes were not leading questions. Concurrent probing was done during the interview, with probes done immediately for each question, rather than at the end of the interview.

For each respondent, the interviewer would read out the question, write down the response, then probe and note down the respondents’ answer after the probe. Some of the probes were already indicated and included in the tool, but the interviewer was at liberty to introduce their own relevant probes as well. Interviewers also observed respondents’ behavior. The interviewer then made a comment about the respondents’ verbal and non-verbal behavior during the interview or about anything else they had observed. Later, the lead author and interviewer made a suggestion for revision of the question where they thought it was necessary.

Interviews lasted an average of 25 min, with privacy ensured by having the interviewers in different rooms and only 1 respondent present at a time. All interviews were conducted in English and audio-recorded. Interviewers filled in some sections of the tool during the interview and then completed the rest after the interviews, supplementing with the audio information. Each respondent provided written informed consent. The study received ethical approval from the higher degrees, research and ethics committee of Makerere University School of Public Health and Uganda National Council for Science and Technology.

### Data analysis

We used multiple ways to “measure” cognition, that is, to identify whether responses given were correct or wrong. Firstly, we relied on our knowledge and experience of the study topic. Secondly, we also compared responses with the instructions in the DHS interviewers’ manual, which explains to the interviewer the intention of each question and the kind of answer expected [22].

This study used a qualitative descriptive approach to analysis [23], where data are presented in a comprehensive and straightforward way, with limited interpretation of participants’ words by the researchers. Preliminary analysis started during the interviews, with interviewers making notes on how the interview was going and suggestions for improvement. These all fed directly into this paper. We present the data in simple, everyday language [23].

### Research team and reflexivity

The specific sections and questions selected from the DHS questionnaire were chosen by DK, the lead author. She used her knowledge and experience from earlier sections of the EN-INDEPTH study, in which she led the qualitative work. Furthermore, she had taken part in training the quantitative data collectors in Iganga Mayuge for the EN-INDEPTH survey, which used the DHS tool (VII). She was therefore aware of the women’s DHS tool, the items therein and what a correct or incorrect answer is. More so, this study built on earlier findings from EN-INDEPTH qualitative work and DK’s PhD, which identified areas of potential comprehension difficulty for women [19].

The research assistants who conducted the cognitive interviews all had vast experience in qualitative field interviews in Uganda and more specifically in the study area. They received prior training on conducting cognitive interviews from DK, the lead author. All four had participated in an earlier related study that involved narratives with people who experienced adverse pregnancy outcomes [24] and one was a data collector in the quantitative EN-INDEPTH survey. Overall, the field team and authors have worked in the field of maternal and newborn health for many years and are thus aware of correct interpretations and common misclassifications of pregnancy outcomes. This combined experience assisted not only in study conceptualization but also data analysis.

### Methodological considerations

Trustworthiness as required in qualitative research is often indicated through the concepts of credibility, transferability, dependability and confirmability [25–27]. In this study, credibility and confirmability were addressed through triangulation of data sources and researchers. Thick description, that is adequate contextual information has been given as part of transferability, while reflexivity addresses both the dependability and confirmability aspects of this study.

## Results

Cognitive interviews were conducted with 20 women, whose socio-demographics are shown in Table 2. Of these, 8 had experienced pregnancy loss, some multiple times and all had living children, ranging from 1 to 7 in number. Half the participants were business women or engaged in trade, five were housewives and there was one secretary, teacher, farmer, tailor and an unemployed participant.

**Table 2:** Socio-demographics of women who participated in cognitive interviews

Characteristic	Number
Age	
20–24	4
25–29	6
30–34	2
35–39	5
40–44	2
45–49	1
Education	
Degree	1
Diploma	4
A level	4
O level	11
Type and number of pregnancy losses experienced	
Miscarriage	3
Abortion (induced)	9
Stillbirth	1
Neonatal death	1

We report findings under the cognitive aspect of comprehension, which as earlier indicated is defined by (i) intent of the question and (ii) meaning of particular terms. We further outline findings under four major birth outcomes, which are (i) births and living children, (ii) neonatal deaths, (iii) miscarriages, abortions and stillbirths and (iv) pregnancy history, in addition to highlighting structural challenges of some questions.

### Intent of the question: what the participants believe the question is asking

Additional file 2 avails more information on the participants’ interpretation of questions versus the intent of the DHS developers, but we provide further explanations in the text below.

#### Births and living children

All participants correctly understood question 201 that asked, “Now I would like to ask about all the births you have had during your life. Have you ever given birth?” Most women repeated it accurately when asked what it meant to them or to repeat it in their own words. This was mostly correctly rephrased as “Have you ever given birth”?

However, most struggled with questions 202 and 204, which ask whether the woman has any sons and daughters she gave birth to who are now living with her (202) or who are alive but do not live with her (204). For instance for 202, some of the women thought they were being asked how many living children they had; the sex of the children; how many children, including non-biological, grandchildren or her husband’s children were living with her and whether she had given away her children to somebody else.

Do I take care of them or I just gave birth and gave them to somebody? (Participant’s interpretation of 202)

You are asking me if the children are still alive, if they are still normal and if not sick, like that (Participant’s interpretation of 202)

The misinterpretations of question 204 were about whether the children were still alive or not and if the parents were looking after them or, like in 202, they had been given to somebody else to look after.

#### Neonatal deaths

Our tool included DHS VIII question 206, which asks about all deaths of offspring of the responding women in the following way: “Have you ever given birth to a boy or girl who was born alive but later died? IF NO, PROBE: Any baby who cried, who made any movement, sound, or effort to breathe, or who showed any other signs of life even if for a very short time?” From this, information on neonatal deaths (occurring during the first 28 days of life) could be extracted.

While many understand this question, others did so partially. For instance, some thought it was about any child who had died and gave answers about stillbirths where a child was born dead or died during delivery.

Have you ever produced a boy or girl who is either alive or dead? (Participant’s interpretation of 206)

In response to question 206, some women asked the interviewer to repeat the question, trying to understand it better. While two women answered that they had lost babies, they did not respond when the interviewer went ahead to ask them to rephrase the question in their own words but instead remained silent.

#### Miscarriages, abortions and stillbirths

We asked the participants question 210, stated as “Women sometimes have a pregnancy that does not result in a live birth. For example, a pregnancy can end in a miscarriage, an abortion, or the child can be born dead. Have you ever had a pregnancy that did not end in a live birth?”.

Overall, the participants understood what the question was looking for. They correctly interpreted that death of a baby could occur, either during the pregnancy or labour. Most of the women said they answered the question with ease because they had been pregnant before and some had suffered these experiences. Nevertheless, there was a challenge with this question because it was long and so some people asked for it to be repeated before they could respond.

Have you ever lost a child during birth? (Participant’s interpretation of 210)

#### Pregnancy history

As part of the cognitive interview we asked question 214, which is the request for pregnancy history: “Now I would like to record all your pregnancies including live births, stillbirths, miscarriages, and abortions, starting with your first pregnancy”. While half the women rephrased this question correctly, interpreting that the interviewer wanted to know about all the pregnancies they had experienced, regardless of the outcome, the other half either completely misunderstood or partially understood.

Examples of misunderstanding included participants thinking that the question was only about babies who died. A few others thought the interviewer wanted to know about the state of their pregnancies and how they progressed or their experiences therein.

It means you are dealing with mothers who have ever given birth or who have undergone delivery or giving birth to babies that have experienced fetal distress, dead or with miscarriages (Participant’s interpretation of 214).

It was not easy because I was trying to understand what you want to record. Do you want the number of children? Do you want what I went through as I am giving birth? So that makes it not to be easy (Participant’s interpretation of 214).

#### Long questions with multiple concepts

Long questions were problematic, especially those that were double barrelled, asking two or more questions within the same sentence but expecting one answer from the respondent. Indeed some questions had more than two parts. For instance question 204: *Do you have any sons or daughters (*statement 1*) to whom you have given birth (*statement 2*) who are alive but do not live with you (*statement 3*)?* Some of the responses to this were answering only one part of the question, or the respondent asked the interviewer to repeat the question so they could understand it better. This was frequent also for other long questions with multiple questions like 210: *Women sometimes have a pregnancy that does not result in a live birth. For example, a pregnancy can end in a miscarriage, an abortion, or the child can be born dead. Have you ever had a pregnancy that did not end in a live birth?*

### Meaning and interpretation of key terms

When we asked question 210, we also inquired from each participant what the terms miscarriage and abortion mean to them, as well as defining what it means to say a child can be born dead. Furthermore, from analysis of questions 210 and 211, we were able to extract more definitions of stillbirth from the participants.

*Miscarriage* Two people described miscarriage as pregnancy loss that occurs when the pregnancy is around 3–4 months. About half of the participants defined miscarriage, partially correctly, as sudden and unintentional loss of the pregnancy, indicating that it came with blood flow and occasionally the fetus. However, most participants did not indicate the time frame in which miscarriage occurred. While a few referred to it as an abortion, they were clear that it was not intentional. There was also a mix up with stillbirths, with one person explaining that a miscarriage can happen early or later on in the pregnancy.

*Abortion*: All participants defined abortion as intentional removal of the pregnancy.

*Stillbirths/Baby born dead* While the term “baby born dead” appeared simple and easy to understand for all participants, only two actually added an element of time, with one saying it would happen at nine months and another describing it as an event that happened during labor, neither of which is factually correct. However, the phrase was easier to clearly define than stillbirths, which some people confused with newborn deaths or mostly understood as death of a child.

In Table 3, we highlight some of the misinterpretations or vague definitions of these terms given by the study respondents, in comparison to the internationally recognized definitions.

**Table 3:** Vague interpretation of key terms compared to international definitions

Miscarriage (International definition) • A spontaneous loss of pregnancy (embryo or fetus) before 22 completed weeks of gestation • Also referred to as spontaneous abortion Source: [34]	Miscarriage: vague or unclear definitions from study participants (quoted verbatim) • Being sure that you are pregnant and all of a sudden blood starts flowing plus the fetus • …a disease which comes when a lady is pregnant for example due to much malaria • When someone is pregnant and fails to reach the age of birth • Loss, losing a pregnancy • Losing a pregnancy when you didn’t want to • Child coming out when it’s not the right time • A baby coming out when it has not made 9 months • Losing pregnancy either early on or later due to diseases like malaria or heavy work
Stillbirths (International definition) Baby born with no signs of life at 22 or more completed weeks of gestation Source: [3]	Stillbirths: vague or unclear definitions from study participants • Babies who have died • Giving birth to a child who has died • “It was somehow hard to answer because I don’t understand that word stillbirths”

## Discussion

In this study, cognitive interviews were conducted to explore how women interpret questions on pregnancy and pregnancy outcomes. Insights have been provided on the framing of various questions. Some questions were easily and correctly understood, especially those that had less technical terms, used common, simple language or did not have multiple parts. For instance, being asked whether one had ever given birth was easy to understand, as was asking about a pregnancy that did not end in a live birth, or a child born dead. Abortion was also easily defined as intentional termination of the pregnancy but further research may be needed to find out if this differs in a less educated group of women in the same setting.

Overall however, most women struggled with interpretation of various questions in the tool. In some cases this was a total misunderstanding but in others it was partial. Among the most challenging areas was the misunderstanding of miscarriage and stillbirths. For instance, although many women defined a miscarriage as unintentional loss of the pregnancy, it was rarely linked to the stage of gestation and in two cases was described as a disease. More so, some definitions of stillbirths were not indicative of gestational age or whether the baby was born alive or not. Stillbirths were also mixed up as miscarriages or newborn deaths. Similar to this study, other studies elsewhere have reported misclassification; for instance in Malawi a study showed that 20% of deaths previously reported in a full birth history survey as neonatal deaths were instead classified as stillbirths during verbal and social autopsy follow up [28]. Our findings are similar to those from Bangladesh, where cognitive interviews conducted on modules of health, nutrition and intra-household relationships reported that although most questions were understood, respondents had difficulty with comprehending some key terms [14].

A previous nutrition coverage study in India reported that surveys noted challenges with long questions that had multiple concepts, with respondents missing the intention of the question [29]. Similarly in our study, long questions with multiple concepts or with two or more questions within the same sentence caused difficulty to some participants. This is likely because the participants were unsure what aspect to respond to and may have suffered with recall of the entire question. For instance, 210 is stated as: Women sometimes have a pregnancy that does not result in a live birth. For example, a pregnancy can end in a miscarriage, an abortion, or the child can be born dead. Have you ever had a pregnancy that did not end in a live birth? The instruction in the DHS VIII interviewer’s manual is to ensure that the full question is read out to the respondent. The subsequent question (211) requires the interviewer to record the number of losses mentioned in 210 together, without differentiation of kind of loss. Therefore while the woman may have mentioned one abortion and one stillbirth, it won’t be recorded that way, thus unclear numbers for each loss.

Our study assessed participants’ comprehension of various survey questions and terms. Among the characteristics of a well-designed survey noted elsewhere is questions that bring out reliable and valid responses from study participants, and inconsistency of questions introduces error [30]. Graesser and others highlight that question misinterpretation by a respondent means an inaccurate answer, so reducing measurement errors requires question modifications to ensure they can be correctly interpreted [30]. They further cite Graesser et al. (1996) who identified 12 frequent difficulties with questions, which are (i) unfamiliar technical terms, (ii) vague terms, (iii) ambiguous nouns, (iv) complex syntax, (v) those that burden the memory, (vi) false or inaccurate presuppositions, (vii) vague question category, (viii) question that falls in more than one category, (ix) unclear question purpose, (x) answer questions differ from what question is asking, (xi) questions for which answers are difficult to recall and (xii) questions for which respondents would probably not know the answer (as cited in [30]).

Our study also observed that despite a participant misinterpreting the question or misunderstanding a particular term, they were predominantly confident about their answers. When asked whether the question was easy or difficult to answer, many of those who had got it wrong said it had been easy to respond to. Others would ask the interviewer to repeat and then either answer correctly or still inaccurately.

This study used the English model version of the DHS VIII questionnaire only because at the time of data collection, the translated *lusoga* (most commonly spoken language in the study area) version was not accessible to the research team. However, a number of women struggled with this interview in English. Understanding of key terms and questions may indeed be influenced by socio-demographics like education and one’s depth of understanding of the interview language. A rapid evaluation to assess respondents interpretation of questions in an HIV indicator survey in Tanzania reported that questions could be misunderstood for different reasons including unfamiliarity with terms or concepts particularly in English, as well as ambiguities and translation challenges [31], similar to the current study.

### Implications for future surveys and how data collection could be improved

Study findings show instances where women misinterpreted the questions but still provided answers. This indicates a high risk of misclassification, especially between miscarriage and stillbirths and between stillbirths and newborn deaths. This potential misclassification has also been acknowledged in other studies [32]. This may result in over reporting of some events and under reporting of others.

Studies have been done elsewhere to assist with formulating questions to be used in surveys, acknowledging that DHS data was incomplete but without an existing way in which to identify new questions [33]. Although not referred to as cognitive interviews, a study on women’s recall of neonatal care in Malawi and Bangladesh, under Saving Newborn Lives program conducted interviews to assess survey questions and concepts and women’s understanding of them. The study findings were partially to inform development of questions for surveys on newborn care in low income settings and recommendations were made around language and instances where there was ambiguity [33].

Our study recommends ways in which some aspects of the tool could be presented differently, some questions rephrased for clarity and general recommendations to improve interviews:

### Introductory explanations of key terms

Firstly, before asking about miscarriage, stillbirths and abortions, it is important to present a commonly understood and standard definition of each of these pregnancy outcomes, which the interviewer should first read and explain to the respondent. This is because at an individual and community level there are variations in understanding the meanings of these terms. However, if the interviewer first described the event of interest well outlined in the tool then it would increase the likelihood that the respondent is talking about the exact outcome defined and not another. In the DHS interviewer manual [22], a brief description of miscarriage, abortion and stillbirth are given but these lack indications of time and / or age and thus are not very clear either.

Furthermore, these key terms need to be appropriately translated to any local languages that will be used in the survey. It will further reduce the chances of the interviewer having to translate according to their own level of knowledge of the language.

### Modifying some problematic questions

In Table 4, suggestions specific to improving certain questions are presented. These focus on improving question clarity, including shorter sentences and avoiding placing different key concepts within the same question.

**Table 4:** Suggested revisions to specific questions

Original question (as it is in DHS VIII)	Suggested revision
202. Do you have any sons or daughters to whom you have given birth who are now living with you?	• Are you living with any of your sons or daughters whom you gave birth to yourself? Probe: Include those who may temporarily be absent e.g. because they are at school, but who otherwise live with you
204. Do you have any sons or daughters to whom you have given birth who are alive but do not live with you?	• Are any of your biological sons or daughters alive but not living with you now?
210. Women sometimes have a pregnancy that does not result in a live birth. For example, a pregnancy can end in a miscarriage, an abortion, or the child can be born dead. Have you ever had a pregnancy that did not end in a live birth?	• Interviewer starts with definition of key terms (miscarriage and abortion; stillbirth here is already indicated as “born dead”) and then proceeds to question. Although this may lengthen the question, at least the data will be more accurate • The question can then be broken into multiple parts with each having its own response category (yes/no), rather than lumping up various outcomes into one question Women sometimes have a pregnancy that does not result in a live birth and I would like to find out from you: • Have you ever had a miscarriage? • Have you ever had an abortion? • Have you ever given birth to a baby who had already died?
214. Now I would like to record all your pregnancies including live births, stillbirths, miscarriages, and abortions, starting with your first pregnancy	• Stillbirth should have already been defined well in question 210

### Adaptations to interviewer training

We also recommend thorough training of interviewers on key terms and their meaning in English and/or the local language, so they can explain them as simply as possible but still accurately, in case the respondent has difficulty with interpretation. Interviewers also need to be trained to identify respondents who are struggling with the language and know when they may need to switch to another language where able.

Additional training to observe non-verbal behavior that may indicate somebody is struggling with understanding the question is necessary. More so, interviewers need to learn to fact-check certain responses, for instance by repeating the response to the respondent, as a means of ensuring that they have given the right or intended answer. Alert listening is key, which can help the interviewer to quickly identify incorrect information regardless of how confident the respondent is with their answers.

### Conducting cognitive interviews rather than basic pre-testing

Although many researchers briefly conduct pre-testing of study tools and make some revisions before data collection, this study recommends conducting cognitive interviews for surveys with questions on pregnancy and pregnancy loss in LMICs where feasible. It is particularly important for those using new tools, introducing new questions or modules, or adapting those used in other contexts to a new population. In this way, the researchers take more time to ensure that the questions are clear and easily understood by potential respondents. Interviewers can also play a role if for instance they are engaged in discussions on how well the respondents understand the tool and are allowed to suggest improvements after the cognitive interviews.

While the DHS VIII tool was the basis for this work, all the above suggestions can be applied to other surveys, in their design and tool preparation or field implementation.

### Future research

This study only conducted a few cognitive interviews in a small geographical area so we cannot certify how widespread the problem is. We recommend studies in other places, with diverse respondents and completion of multiple cycles of revising the tools until participants understand them better, as often happens in cognitive interviews. It is also important to explore interviewers’ own understanding of the tool and survey questions, as well as to conduct cognitive interviews on the translated tools.

### Strengths and limitations

This study is unique in conducting cognitive interviews on the reproduction section of the DHS VIII tool with women in Uganda. It provides new insights into women’s comprehension of questions and the implications this has for data accuracy. The study was limited by the fact that only one round of the cognitive interviews was conducted, unlike some other studies that do repeat rounds, improving the tool with the respondents’ feedback until there is a level of satisfaction with it [13]. Additionally, only participants with secondary school education were included, who are not representative of the wider population.

## Conclusion

Population surveys may misclassify, over or under report events around pregnancy and pregnancy outcomes. The survey interview tool and questions therein play a critical role in collection of accurate data. Questions can be simplified through improved sentence structure, that is, short and simple sentences that are not double barreled. Additionally, interviewers should begin with a standard definition of key terms and ensure that respondents understand these. More so, interviewer training should be modified to ensure they also thoroughly understand key terms. We recommend cognitive interviews while developing survey tools or when applying them in new populations where they have not been used before, beyond basic pre-testing. Improving respondents’ comprehension and thus response accuracy will increase reporting and data quality.

### Supplementary Information

**Additional file 1. **Complete cognitive interview questionnaire used in the study.

**Additional file 2. **Participants’ interpretation of questions in comparison to DHS VIII instructions.

## Data Availability

The datasets (completed cognitive interviews) generated and analyzed during the current study are not publicly available due to the need to protect privacy of participants but are available from the corresponding author on reasonable request.

## Abbreviations

APOs: Adverse pregnancy outcomes
DHS: Demographic and health survey
EN-INDEPTH: Every Newborn—International Network for the Demographic Evaluation of Populations and Their Health
HDSS: Health and demographic surveillance system
IMHDSS: Iganga Mayuge health and demographic surveillance system
LMIC: Low and middle income countries
NMR: Neonatal mortality rate
UNICEF: United Nations Children’s Fund
VHTs: Village health teams
