# Supplementary material for: Exploring women’s interpretations of survey questions on pregnancy and pregnancy outcomes: cognitive interviews in Iganga Mayuge, Uganda
Source: Reprod Health. 2024 Jan 29;21:14. doi: 10.1186/s12978-024-01745-w (PMC10826263; doi:10.1186/s12978-024-01745-w)
Supplement: Supplementary file 1 — Additional file 1. Complete cognitive interview questionnaire used in the study. [file 12978_2024_1745_MOESM1_ESM.pdf]

Additional file 1: Cognitive interview questionnaire: Adapted reproduction section of woman's questionnaire – DHS VIII, English

Interviewer name ___ ___ ___ ___ Respondent ID ___ ___ ___ ___ Interview date: __ __ / __ __ / ___ ___ (*DD/MM/YY*)

Time interview started: : ☐☐ ☐☐

Section A – interview questions from DHS

**Original question**	**Response categories**	**Probes (Pre-set & by interviewer)**	**Respondent's answer after probe**	**Interviewer's comment (on verbal & non-verbal behavior)**	**Suggested revision**
201. Now I would like to ask about all the births you have had during your life. Have you ever given birth?	Yes<br><br>No *(If no, skip to 206)*	• Can you repeat the question in your own words?<br>• *Any other probe by interviewer*			
202. Do you have any sons or daughters to whom you have given birth who are now living with you?	Yes<br><br>No *(If no, skip to 204)*	• What does this question mean to you?<br>• *Any other probe by interviewer*			
204. Do you have any sons or daughters to whom you have given birth who are alive but do not live with you?	Yes<br><br>No *(If no, skip to 206)*	• What does this question mean to you?<br>• *Any other probe by interviewer*			
206. Have you ever given birth to a boy or girl who was born alive but later died?<br><br>IF NO, PROBE: Any baby who cried, who made any	Yes<br><br>No *(If no, skip to 209 to calculate total births)*	• Can you repeat the question in your own words?<br>• How easy or difficult did you find this question to answer? Why do you say that?			

movement, sound, or effort to breathe, or who showed any other signs of life even if for a very short time?		• *Any other probe by interviewer*<br>(e.g. I noticed you hesitated before you answered – what were you thinking about?)			
207.<br>a) How many boys have died?<br>b) And how many girls have died?<br><br>IF NONE, RECORD '00'.	a) Boys dead………<br><br>b) Girls dead…………	• How easy or difficult did you find this question to answer? Why do you say that?<br>• Were you comfortable answering this question? Why or why not?<br>• *Any other probe by interviewer*			
209. Just to make sure that I have this right: you have had in TOTAL _____ births during your life. Is that correct?					
210. Women sometimes have a pregnancy that does not result in a live birth. For example, a pregnancy can end in a miscarriage, an abortion, or the child can be born dead. Have you ever had a pregnancy that did not end in a live birth?	Yes<br><br>No *(If no, skip to 214)*	• Can you repeat the question in your own words?<br>• What does miscarriage mean to you?<br>• What does abortion mean to you?<br>• What does it mean to you when I say a child can be born dead?<br>• How easy or difficult did you find this question to			

		answer? Why do you say that?<br>• *Any other probe by interviewer*			
211. How many miscarriages, abortions, and stillbirths have you had?	Pregnancy losses………………….	• Can you repeat the question in your own words?<br>• How easy or difficult did you find this question to answer? Why do you say that?<br>• Were you comfortable answering this question? Why or why not?<br>• *Any other probe by interviewer*			
214. Now I would like to record all your pregnancies including live births, stillbirths, miscarriages, and abortions, starting with your first pregnancy.		• What does this question mean to you?<br>• Was this hard or easy? Why do you say so?<br>• *Any other probe by interviewer*<br>(e.g. I noticed you hesitated before you answered – what were you thinking about?)			
232. Are you pregnant now?	Yes<br>No<br>Unsure<br>*(If No or Unsure, end the interview)*	• How sure of your answer are you?<br>• How easy or difficult did you find this question to answer? Why do you say that?			

		• Were you comfortable answering this question? Why or why not?<br>• How would you say that question yourself?<br>• *Any other probe by interviewer*<br>(e.g. I noticed you hesitated before you answered – what were you thinking about?)			
233. How many weeks or months pregnant are you?	Weeks……………..<br><br>Months……………	• How sure of your answer are you?<br>• How did you arrive at that answer?<br>• Was it easy or difficult to answer this question? Why do you say that?<br>• How would you say that question yourself?<br>• *Any other probe by interviewer*			

Thank subject for her time and end the interview

Time interview ended : ☐☐:☐☐

B. Other areas for the interviewer to fill in after interview

Please describe any other problems you observed during the interview

- Where respondent had difficulty answering a question
- Were they hesitant over some questions?
