# Supplementary material for: Exploring women’s interpretations of survey questions on pregnancy and pregnancy outcomes: cognitive interviews in Iganga Mayuge, Uganda
Source: Reprod Health. 2024 Jan 29;21:14. doi: 10.1186/s12978-024-01745-w (PMC10826263; doi:10.1186/s12978-024-01745-w)
Supplement: Supplementary file 2 — Additional file 2. Participants’ interpretation of questions in comparison to DHS VIII instructions. [file 12978_2024_1745_MOESM2_ESM.pdf]

Additional file 2: Participants' interpretation of questions in comparison to DHS VIII instructions

**Original question (as it is in DHS VIII)**	**Participants' varying interpretations of the question**	**Intention of the question (as indicated in DHS interviewer's manual instructions)**
202. Do you have any sons or daughters to whom you have given birth who are now living with you?	• How many sons and daughters do I have?<br>• Do I take care of them or I just gave birth and gave them to somebody?<br>• It means I can have people at home but not my biological children<br>• It means that children you have given birth to, who are still alive<br>• It means that do I have any kids I am living with? Not so?<br>• You are asking me if the children are still alive, if they are still normal and if not sick, like that<br>• To my understanding you are asking me that the husband whom I have, do I have any child of his?<br>• Do I have grand children living with me?<br>• The question is meaning, how many female children and male children am I having? Daughters and sons, it means males and female sex	The sons and daughters being considered are her OWN biological children who live with her in her household (which will usually be the household in which the interview is being held, except for women who are visitors)
204. Do you have any sons or daughters to whom you have given birth who are alive but do not live with you?	• They were asking me about those sons I gave birth to and if they are there or not<br>• As a parent, are you taking care of the children?<br>• Do I have any children outside my family that I am living with now?<br>• Children you have produced but you are not caring for them and have given to someone else<br>• Do I have my sons whom I live with?	These questions refer to the respondent's sons and daughters who are alive but not living with her. For example, they may be living with a relative, may be staying at a boarding school, may have been given up for adoption, or may be grown-up children who have left home.
206. Have you ever given birth to a boy or girl who	• Have I ever produced a child and it died?<br>• Have I ever lost a baby boy or girl?	These questions on children who have died are extremely important and are among the most

<table>
<tr><td>was born alive but later died?

IF NO, PROBE: Any baby who cried, who made any movement, sound, or effort to breathe, or who showed any other signs of life even if for a very short time?</td><td>You said that have you ever lost a child whether still young or during birth?
Have you ever produced a boy or girl who is either alive or dead?</td><td>difficult on which to obtain accurate data. Some respondents may fail to mention children who died very young, so if a woman answers NO, it is important to probe by asking, “Any baby who cried, who made any movement, sound, or effort to breathe, or who showed any other signs of life even if for a very short time?”</td></tr>
<tr><td>210. Women sometimes have a pregnancy that does not result in a live birth. For example, a pregnancy can end in a miscarriage, an abortion, or the child can be born dead. Have you ever had a pregnancy that did not end in a live birth?</td><td>Some misconstrued interpretations included the following:
I have heard that have you ever had a pregnancy that did not end in whether the baby died…
Have you ever lost a child during birth?</td><td>Now, in Q. 210, we want to know whether the respondent had any pregnancies that did not result in a live birth. To ensure that none are missed, the question specifically mentions the three ways a pregnancy may not result in a live birth:
Miscarriage: a woman’s pregnancy ended early and involuntarily
Abortion: the woman voluntarily ended a pregnancy
Stillbirth: the woman gave birth to a child that showed no signs of life (was born dead)
Make sure to read the full question to the respondent</td></tr>
<tr><td>214. Now I would like to record all your pregnancies including live births, stillbirths, miscarriages, and abortions, starting with your first pregnancy</td><td>While many understood the questions well, a few women misinterpreted them, for instance:
It means you are dealing with mothers who have ever given birth or who have under gone delivery or giving birth to babies that have experienced fetal distress, dead or with miscarriages
You want to know my life status during my pregnancy</td><td>Begin the section by informing the respondent that we would like to record all of her pregnancies, from all marriages and unions, including live births, stillbirths, miscarriages, and abortions.</td></tr>
</table>

	• You want to know all about my pregnancies. I have said that my pregnancies are okay because I produce my babies when they are alive<br>• It means the day I got my first pregnancy, when and which date and which year?<br>• It was not easy because I was trying to understand what you want to record. Do you want the number of children? Do you want what I went through as I am giving birth? So that makes it not to be easy	
